# Development of Multi-Compartment 3D-Printed Tablets Loaded with Self-Nanoemulsified Formulations of Various Drugs: A New Strategy for Personalized Medicine

**DOI:** 10.3390/pharmaceutics13101733

**Published:** 2021-10-19

**Authors:** Tarek A. Ahmed, Raed I. Felimban, Hossam H. Tayeb, Waleed Y. Rizg, Fuad H. Alnadwi, Hanadi A. Alotaibi, Nabil A. Alhakamy, Fathy I. Abd-Allah, Gamal A. Mohamed, Ahmed S. Zidan, Khalid M. El-Say

**Affiliations:** 1 Department of Pharmaceutics, Faculty of Pharmacy, King Abdulaziz University, Jeddah 21589, Saudi Arabia; wrizq@kau.edu.sa (W.Y.R.); halalotaibi@kau.edu.sa (H.A.A.); nalhakamy@kau.edu.sa (N.A.A.); kelsay1@kau.edu.sa (K.M.E.-S.); 2 Center of Research Excellence for Drug Research and Pharmaceutical Industries, Pharmaceutical Technology Unit, King Abdulaziz University, Jeddah 21589, Saudi Arabia; 3 Center of Innovation in Personalized Medicine (CIPM), 3D Bioprinting Unit, King Abdulaziz University, Jeddah 21589, Saudi Arabia; faraed@kau.edu.sa; 4 Department of Medical Laboratory Technology, Faculty of Applied Medical Sciences, King Abdulaziz University, Jeddah 21589, Saudi Arabia; hhtayeb@kau.edu.sa; 5 Center of Innovation in Personalized Medicine (CIPM), Nanomedicine Unit, King Abdulaziz University, Jeddah 21589, Saudi Arabia; 6 Department of Nuclear Engineering, Faculty of Engineering, King Abdulaziz University, Jeddah 21589, Saudi Arabia; fnadwi@kau.edu.sa; 7 Department of Pharmaceutics and Industrial Pharmacy, Faculty of Pharmacy, Al-Azhar University, Cairo 11651, Egypt; fibrahim@icbr.info; 8 Department of Natural Products and Alternative Medicine, Faculty of Pharmacy, King Abdulaziz University, Jeddah 21589, Saudi Arabia; gahussein@kau.edu.sa; 9 Department of Pharmaceutics and Industrial Pharmacy, Faculty of Pharmacy, Zagazig University, Zagazig 44519, Egypt; Ahmed.Zidan@fda.hhs.gov

**Keywords:** 3D-printed tablets, rosuvastatin, glimepiride, SNEDDS, curcumin oil, personal medicine

## Abstract

This work aimed to develop a three-dimensional printed (3DP) tablet containing glimepiride (GLMP) and/or rosuvastatin (RSV) for treatment of dyslipidemia in patients with diabetes. Curcumin oil was extracted from the dried rhizomes of *Curcuma longa* and utilized to develop a self-nanoemulsifying drug delivery system (SNEDDS). Screening mixture experimental design was conducted to develop SNEDDS formulation with a minimum droplet size. Five different semi-solid pastes were prepared and rheologically characterized. The prepared pastes were used to develop 3DP tablets using extrusion printing. The quality attributes of the 3DP tablets were evaluated. A non-compartmental extravascular pharmacokinetic model was implemented to investigate the in vivo behavior of the prepared tablets and the studied marketed products. The optimized SNEDDS, of a 94.43 ± 3.55 nm droplet size, was found to contain 15%, 75%, and 10% of oil, polyethylene glycol 400, and tween 80, respectively. The prepared pastes revealed a shear-thinning of pseudoplastic flow behavior. Flat-faced round tablets of 15 mm diameter and 5.6–11.2 mm thickness were successfully printed and illustrated good criteria for friability, weight variation, and content uniformity. Drug release was superior from SNEDDS-based tablets when compared to non-SNEDDS tablets. Scanning electron microscopy study of the 3DP tablets revealed a semi-porous surface that exhibited some curvature with the appearance of tortuosity and a gel porous-like structure of the inner section. GLMP and RSV demonstrated relative bioavailability of 159.50% and 245.16%, respectively. Accordingly, the developed 3DP tablets could be considered as a promising combined oral drug therapy used in treatment of metabolic disorders. However, clinical studies are needed to investigate their efficacy and safety.

## 1. Introduction

The ability to tune or tailor a formulation by flexible control of drug formulation, dose and physicochemical properties has a great impact on the healthcare sector especially for pediatric and geriatric patients. This concept is of great importance particularly when the physiology, drug response, and genetic profile of patients are considered [1,2]. The current medical treatment strategy is based on the concept of “one size fits all” where the same drugs are administered at the same doses and same frequencies [3]. Treatment of different patients using this “one size fits all” strategy has revealed varied responses. Therefore, an effective technology such as three-dimensional printing which offers the controllable and tunable production of patient-customized and personalized dosage forms is needed [4]. This will help in improving the quality of healthcare of chronic patients and in reducing the effort, time, and the financial obstacles involved during the manufacturing of pharmaceuticals. Personalized medicine is a promising strategy that is more safe, efficacious, cost effective, and leads to enhancement in patient compliance [5].

3D bio-printing has been utilized in tissue engineering and different pharmaceutical applications [6]. In this process, 3D printers are used to create the intended structure. Depending on the purpose of the application of the biomaterials, cells or growth factors are used to develop biomedical parts. The major disadvantages of this process include the deformation of cellular structure and loss of cellular viability due to the pressure used to expel the bioink, the fact that the process is time consuming, and the limited number of materials compatible for use with some types of printers (such as the stereolithography method) [7]. 3D printing of pharmaceutical dosage forms holds a great promise in the fabrication of active pharmaceutical ingredients (APIs) toward the personalization of medications. This is achieved through tuning the printing process parameters which include the tablet’s size and layer thickness to control dosage form and release. The use of 3D printing technology in the development of personalized medicine and producing individualized drug dosage forms in the pharmaceutical industry could be useful [8]. For example, the formulation of a multi-drug single and one daily dose tablet, with appropriate release profiles, provides an attractive alternative manufacturing method. 3D printing has also been employed in the development of dosage forms containing multiple drugs of different release profiles [9].

Many studies have shown the effect of bioactive constituents extracted from *Curcuma longa* on the treatment of inflammation, neurodegenerative disorders, cancer, and diabetes [10,11,12]. Activation of immune response, antibacterial and antiviral effects, anthelminthic, anti-Alzheimer, antioxidant and antinociceptive activities have also been mentioned [13,14]. There is also an increasing interest in curcumin oil for its protective roles in cardiovascular disorders by lowering the total blood cholesterol and low-density lipoprotein levels [15]. The essential oil extracted from the rhizomes of *Curcuma longa* has been reported to have favorable characteristics for human consumption [16]. Self-nanoemulsifying drug delivery system (SNEDDS) is a promising approach to enhance the absorption of poorly water-soluble drugs [17]. SNEDDS is an isotropic mixture of oil, surfactant, and cosurfactants that is used to encapsulate or deliver cargos including hydrophobic drugs. SNEDDS enables the administration of hydrophobic drugs as a unit dosage form for oral treatment [18].

Patients with diabetes have a two to four-fold higher risk of cardiovascular disorders than that among non-diabetic patients [19]. There is a demand for administration of a lipid lowering agent and antidiabetic drug simultaneously, since the main cause of mortality in diabetic patients is the cardiovascular disorders [20]. Therapeutic regimes using glimepiride (GLMP) and rosuvastatin (RSV) are currently available as a separate oral dosage form to treat diabetes and hyperlipidemia. A combination of both drugs (GLMP and RSV) in a single dosage form could be helpful, especially if curcumin oil is added since the latter has been reported to have roles in cardiovascular disorders (as mentioned above). GLMP is a hydrophobic anti-diabetic agent of the sulfonylurea group [21]. GLMP has a biological half-life of approximately 5 h. RSV is the most potent and widely prescribed statin member that decreases low-density lipoprotein cholesterol and is therefore prescribed to prevent cardiovascular disorders [22]. RSV is characterized by its limited solubility in the gastrointestinal fluids and is subjected to extensive first-pass metabolism, the effects that result in a limited drug oral bioavailability (approximately 20%) [23,24]. To manage dyslipidemia in diabetic patients, both drugs (GLMP and RSV) should be administered concomitantly [20].

The use of 3D printing to formulate pharmaceutical dosage forms still remains in its early stages and this technique is facing many challenges related to regulatory aspects, quality control, and technical issues. More investigations in this area will open the door for the pharmaceutical sector to embrace this technology, an effect that will have a positive impact on personalized medicine and the healthcare system. In this work, we have investigated the development of 3DP multi-compartment oral tablets containing GLMP and RSV loaded in a SNEDDS formulation. The pharmacokinetics of the prepared tablets were studied and compared to marketed drug products to investigate the usefulness of this technology for personalized medicine.

## 2. Materials and Methods

### 2.1. Materials

Glimepiride (GLMP) and rosuvastatin (RSV) were kindly gifted by the Saudi Pharmaceutical Industries & Medical Appliances Corporation (SPIMACO) (Alqasim, Saudi Arabia) and the Saudi Arabian Japanese Pharmaceuticals Co., Ltd. (SAJA) (Jeddah, Saudi Arabia), respectively. Microcrystalline cellulose (Avicel) PH-101 was procured from Winlab laboratory chemicals (Leicestershire, UK). Hydroxypropyl methyl cellulose (HPMC) 4000 cp was obtained from Spectrum Chemical Manufacturing Corporation (Gardena, CA, USA). Lactose anhydrous, Polyethylene glycol (PEG) 400, Tween 80, Polyvinyl pyrrolidone (PVP) with a molecular weight of 360,000 Da (K90), Methocel^®^ A15 LV, 27.5–31.5% methoxyl basis were purchased from Sigma-Aldrich Inc. (St. Loius, MO, USA). Croscarmellose sodium (Ac-di-sol) was obtained from Biosynth International, Inc., (San Diego, CA, USA).

### 2.2. Plant Material and Oil Extraction

*Curcuma longa Linn* (Turmeric) rhizomes (Zingiberaceae) were obtained from a local market in Jeddah, Saudi Arabia. The plant’s authentication was established by Dr. Emad Al-Sharif, professor of plant ecology at the Faculty of Science & Arts, King Abdulaziz University and a voucher specimen (no. CL1442) was kept in the herbarium at Faculty of Pharmacy, KAU.

Extraction and analysis of the oil were achieved as previously reported [25]. Briefly, the dried rhizomes (3.5 kg) were grinded and extracted several times with *n*-hexane (8 L × 6) at room temperature. The solvent was distilled off under reduced pressure below 50 °C to yield an orange yellow odoriferous viscous oil (45 g).

### 2.3. Development of Curcuma Oil Based SNEDDS

The oil of *curcuma longa* (X_1_) was used to develop different SNEDDS formulations utilizing Tween 80 (X_2_) and PEG 400 (X_3_) as surfactant and cosurfactant, respectively. Mixture experimental screening design of extreme vertices was used to develop a SNEDDS formulation of a minimum droplet size utilizing the statistical package Statgraphics^®^ Centurion XV Software, Version 15.2.05 StatPoint Technologies, Inc. (Warrenton, VA, USA). A total of thirteen runs were proposed after identifying the range of the studied factors. Each run contains a specified weight of the three components that was always added up to 100%. Tween 80 and PEG 400 were used as they have been successfully utilized, in our previously published work, in the development of SNEDDS with different oils such as sefsol, linoleic acid, olive oil, oleic acid and isopropyl myristate [26,27,28]. The composition of the prepared SNEDDS formulations is illustrated in Table 1. Briefly, 1 g of each SNEDDS formulation was prepared in a screw cap vials by accurately weighing the specified amount of each component. Each formulation mixture was subjected to vortex until a homogenous dispersion was obtained.

Known weight of each formulation was added to a specified volume of distilled water, in a ratio of 1:10 (*w*/*v*), on a magnetic stirrer until a homogenous yellowish nanoemulsion was obtained. Malvern Zetasizer Nano ZSP, Malvern Panalytical Ltd. (Malvern, UK) was used to evaluate the droplet size and polydispersity index (PDI) of the prepared nanoemulsions. Measurement for each sample was performed in triplicate.

Data obtained for the droplet size were analyzed using Statgraphics^®^ Centurion XV Software, Version 15.2.05 StatPoint Technologies, Inc. (Warrenton, VA, USA). The SNEDDS formulation with the minimum droplet size was identifying. This formulation was prepared and characterized as mentioned above.

### 2.4. Preparation of Pastes

Two types of gel formulations were prepared using HPMC (4% *w*/*v*) as a gelling agent. SNEDDS-based and non-SNEDDS gel formulations were prepared according to the formulation composition depicted in Table 2.

Non-SNEDDS gels were prepared by dispersing known weight of RSV or GLMP in distilled water over a magnetic stirrer. HPMC was added gradually to the mixture while stirring. The obtained medicated polymeric formulations were left over night at 4 °C in a refrigerator to allow complete swelling of the HPMC particles and formation of viscous gels.

SNEDDS-based gels were prepared by adding known weight of RSV or GLMP into 2 g of the prepared SNEDDS formulation. The medicated SNEDDS formulation was added to 18 mL of distilled water over a magnetic stirrer. HPMC was added gradually to the mixture with continuous stirring. The medicated polymeric mixture was left over night in the refrigerator. A non-medicated (drug free) SNEDDS-based gel matrix formulation was also prepared.

A powder mixture of Avicel (20% *w*/*w*), PVPK90 (10% *w*/*w*), lactose (10% *w*/*w*), methocel (5% *w*/*w*), and Ac-Di-Sol (5% *w*/*w*) was well-blended and transferred to a mortar containing the prepared gel matrix. Mixing of the ingredients was continued until a smooth homogenous paste was obtained. PVPK90, lactose and methocel were used as adsorbent for the SNEDDS formulation. Avicel was used as an insoluble ingredient and Ac-Di-Sol was used as a disintegrant.

### 2.5. Rheological Characterization

A Kinexus oscillation rheometer (Malvern Instruments Ltd., Worchestershire, UK) was utilized to investigate the rheological behavior of the prepared formulations (F1–F6) using the rSpace software package. The apparatus is equipped with a parallel plate geometry with a gap size of 1 mm. Rheograms for the relation between viscosity and shear rate were obtained as a function of shear rate in the range 0.01 to 1000 s^−1^ at 20 °C. Creep recovery mode has been employed to investigate the viscoelastic behavior of the pastes by applying a shear stress “strain” for a definite period followed by releasing. To identify the strain after which the flow for each paste starts an amplitude strain sweep was used by applying an oscillatory strain. The following power law relation was used to describe the reduction in viscosity as a function of shear rate:

Ƞ = K·Y^(n−1)^ (1)

where: Ƞ is the apparent viscosity, Y is the shear rate (s^−1^), K is the consistency constant, n is the flow behavior index. In the pseudoplastic regime n ≤ 1 (typically n = 0.4 to 0.7) [29].

### 2.6. 3D-Printing of Tablets

The prepared paste formulations were developed into 3DP tablets using a REGEMAT3D V1 BioPrinter (REGEMAT Inc., Granada, Spain) utilizing a computer-aided design (CAD) modelling software (REGEMAT 1.4.9 Designer). The pastes were freshly prepared, loaded separately into a Syringe of 5 cc and extruded through a 0.58 mm printing nozzle with a flow speed of 2.6 mm/s and infill speed of 10 mm/s. The printing nozzle was set to move in a vertical and horizontal directions to place the paste in the selected area of the building plate at room temperature. The printing process was carried out layer-by-layer for building of the 3DP tablet structure. The average time needed to complete the printing process for each tablet was approximately 10 min. All tablets (F1–F5) were printed as a cylinder of 15 mm diameter utilizing eight layers. The printed tablets were kept in a vacuum dryer at 40 °C for 24 h to allow fusion of the layers and complete drying of the tablets. Finally, the dried tablets were stored in a hermetically sealed container until further analysis. Tablets of formulation six “F6” were printed using an equal amount of F4 and F5 pastes formulations. The concentration of GLMP and RSV in these formulations can be modified according to the personal need.

### 2.7. Characterization of the 3DP Tablets

The prepared dried 3DP tablets (F1–F6) were characterized for weight variation, thickness, diameter, friability, drug(s) content, and in vitro drug release. All tests were conducted according to the requirements stated in the United States Pharmacopeia for quality control tests of tablets [30]. The average weight of ten tablets, from each formulation, was estimated using a Mettler Toledo AJ100 electric balance (Greifensee, Switzerland). The thickness and diameter (n = 10) were determined using Mitutoyo dial thickness gauge (Kawasaki, Japan). Friability (n = 10) of the prepared tablets was evaluated using Erweka Friabilator type PTF1, Pharma-test (Hainburg, Germany). It was calculated as a fraction of the weight of the original tablets after allowing the studied tablets to rotate in the test apparatus for 4 min at 25 rpm.

The concentration of GLMP in F2 and F4 tablet formulations, and the concentration of RSV in F3 and F5 tablet formulations were determined by placing separately ten tablets from each formulation in a separate glass bottles containing 100 mL of 0.1 N NaOH. The bottles were left overnight in a shaking water bath, Model 1031; GFL Corporation (Burgwedel, Germany), at 25 °C. The content of each bottle was thoroughly homogenized using UltraTurax, IKA^®^ T18 basic Homogenizer (Campinas, Brazil) for 10 min and subjected to filtration through filter paper. The filtrate was further diluted and the concentration of GLMP or RSV was determined spectrophotometrically against a blank of F1. Tablets of F6 were also subjected to the same procedure except that the concentration of both drugs was determined using the simultaneous equations that will described below.

The in vitro drug release of the prepared 3D-printed tablet formulations was studied using the paddle type USP dissolution test apparatus (type II), DT 700 LH device, Erweka GmbH DT 700 (Heusenstamm, Germany). The test was carried out in 900 mL of distilled water containing 0.1% sodium lauryl sulfate at 37 °C [31,32]. The paddle speed was adjusted at 75 rpm. Samples of 5 mL, with immediate replacement, were withdrawn at 0.25, 0.5, 1, 2, 3, 4, 5, 6 and 12 h. The collected samples were filtered and assayed for drug(s) content, against blank of F1. Profiles for GLMP and RSV release were constructed. The experiment was conducted in triplicate.

GLMP and RSV were quantified in F2, F3, F4 and F5 tablets spectrophotometrically at 228 nm and 241 nm, respectively. For simultaneous quantification of both drugs in F6 tablets, a simple spectrophotometric method has been used as previously described, except for slight modification [33]. A standard calibration curve that relates the absorbance and the concentration, for each drug, was constructed and the equation of the line which fits to the data was generated. The absorptivity values for GLMP and RSV were determined. Equations (2) and (3) were used to estimate the concentration of both drugs in the prepared combined standard solutions and in the pharmaceutical formulations.

Concentration of RSV = (A_2_ ay_1_ − A_1_ ay_2_)/ (ax_2_ ay_1_ − ax_1_ ay_2_) (2)

Concentration of GLMP = (A_1_ ax_2_ − A_2_ ax_1_)/ (ax_2_ ay_1_ − ax_1_ ay_2_) (3)

where A_1_ is the absorbance of the sample at 241 nm, A_2_ is the absorbance of the sample at 228 nm, ax_1_ is the absorptivity of RSV at 241 nm, ax_2_ is the absorptivity of RSV at 228 nm, ay_1_ is the absorptivity of GLMP at 24 nm and ay_2_ is the absorptivity of GLMP at 228 nm.

### 2.8. Solid-State Physicochemical Characterization

Nicolet Is10 Fourier transform infrared (FT-IR) spectrometer of Thermo Scientific, Inc. (Waltham, MA, USA) and D/max 2500; Rigaku, powder X-ray diffractometer (Tokyo, Japan) were used to investigate the FT-IR spectrum and the crystalline nature of GLMP, RSV and tablet formulations F4, F5, and F6. Preparation of the samples and the condition of the experiments were conducted as previously described [34].

### 2.9. Scanning Electron Microscope (SEM)

To investigate the prepared tablets inner and surface structure, SEM images for SNEDDS-based (F1) and non-SNEDDS (F2) tablets were taken utilizing Philips XL30 SEM (Eindhoven, Netherlands). Samples from the studied tablet surface and inner layers were prepared using a surgical scalpel. The prepared samples were mounted onto aluminum stubs and sputter-coated with gold. Images were taken at an accelerating voltage of 10 kV.

### 2.10. Pharmacokinetics Study

The pharmacokinetics of GLMP and RSV from the prepared 3DP tablets containing combined drug medication (F6) were studied and compared to marketed products. A single dose one-period parallel design, utilizing male Wistar rats having an average weight of 200–250 g, was used. Animals were classified into three groups (n = 6). Group I received the 3DP printed tablets containing GLMP and RSV combination (F6). Group II was given the marketed GLMP tablets (Amaryl^®^ of Hoechst Marion Roussel, Stockholm, Sweden). Group III administered marketed RSV tablets (Rosavi^®^ of the Saudi Arabian Japanese Pharmaceuticals Co. Ltd., Jeddah, Saudi Arabia). Before treatment, animals were kept in a temperature-controlled closed area with a 12-h light/darkness cycles for one week with free access to water and food. GLMP and RSV doses of 10 mg/kg and 20 mg/kg, respectively, were used [35,36]. The prepared 3DP tablets and the marketed products were crushed and suspended in 1% carboxymethyl cellulose to prepare a drug suspension suitable for oral animal administration through a gastric tube. Blood samples were collected from the studied animals through retro-orbital puncturing at 1, 2, 4, 6, 12, and 24 h for GLMP while, the blood sampling was conducted to 72 h for RSV. The collected plasma samples were immediately separated by centrifugation at 6000 rpm for 10 min and stored at −20 °C.

The protocol for this study received approval from the Research Ethics Committee, Faculty of Pharmacy, King Abdulaziz University, KSA (Reference No. 1021442). The study was conducted following the European Medicines Agency (EMA), International Conference on Harmonization (ICH), Good Clinical Practice (GCP), and Food and Drug Administration (FDA) guidelines.

For determination of GLMP, calibration standards were prepared from blank plasma samples using GLMP and metformin (internal standard) methanolic stock solutions. The plasma concentration of GLMP in the unknown and the prepared calibration standards was determined using a Perkin Elmer high-performance liquid chromatograph (HPLC) equipped with variable wavelength UV detector and auto sampler. Phenomenex, RP Hi-Q-Sil C18 column (250 × 4.6 mm, 5 µm, Phenomenex Inc., Torrance, CA, USA) was used for separation. The mobile phase consisted of (64:36) acetonitrile and potassium dihydrogen orthophosphate (0.02 M) adjusted to pH 3.5. The injection volume used was 30 µL. The flow rate of the mobile phase was adjusted at 1 mL/min. The UV detector was set at 230 nm. For extraction and preparation of the GLMP samples; 1 mL of acetonitrile-methanol (1:1) mixture was added to each sample. The mixture was vortexed for 1 min and centrifuged for 10 min at 5000 rpm. The organic phase was separated and evaporated to dryness under constant stream of nitrogen at 50 °C. The residue was reconstituted in 80 µL of the mobile phase and a volume of 30 µL was injected. The condition employed for quantification of GLMP in the plasma samples was conducted as previously mentioned except for slight modification [37].

For RSV determination, atorvastatin was used as an internal standard. Quantification of RSV in the calibration standards and the unknown samples was carried out using the same apparatus and condition mentioned above for GLMP except that the mobile phase consisted of acetonitrile and 0.1% phosphoric acid (58:42) and the UV detector was adjusted at 240 nm. Extraction and separation of RSV were achieved as described above for GLMP except that the residue was reconstituted in 80 µL of acetonitrile and 0.1% phosphoric acid (58:42). The method used for analysis of RSV in the plasma samples was conducted utilizing the condition previously reported by Ahmed et al. [36].

PKsolver (An add-in program for pharmacokinetic data) was used to calculate the studied pharmacokinetic parameters utilizing a non-compartmental extravascular pharmacokinetic model.

### 2.11. Statistical Analysis

The obtained data for the pharmacokinetics of GLMP and RSV were expressed as mean ± SD and statistically analyzed utilizing the GraphPad Prism 8 software (GraphPad Inc., La Jolla, CA, USA).

## 3. Results and Discussion

### 3.1. Mixture Experimental Screening Design

Based on the extreme vertices mixture design, thirteen SNEDDS formulations were prepared. The order of the experimental runs has been fully randomized to provide protection against the effects of lurking variables. The studied SNEDDS formulations showed a particle size between 93–359 nm, as displayed in Table 1, and a PDI of 0.234–0.667. Different models are available to fit the data which include the linear, quadratic, special cubic, and the cubic model. The linear model consists of first-order terms for each of the components. The quadratic model adds cross-products between pairs of components. The special cubic model adds terms involving products of the three components. The cubic model adds other third-order term. Each model is shown with a *p-value* which tests whether that model is statistically significant when compared to the mean square for the term below. Normally, we would select the most complicated model with a *p-value* less than 0.05, assuming that we are operating at the 95.0% confidence level. According to this criterion, it appears that the special cubic model (*p-value* = 0.0158) is adequate for the data. The quadratic and cubic models showed *p-values* of 0.3457 and 0.5684, respectively.

Analysis of variance (ANOVA) for the currently selected cubic model revealed that the *p-value* for this model is less than 0.05, and there is a statistically significant relationship between the droplet size and the components at the 95.0% confidence level. The R-Squared statistic indicates that the model as fitted explains 99.62% of the variability in droplet size. The adjusted R-squared statistic, which is more suitable for comparing models with different numbers of independent variables, is 98.48%. Correlation matrix for coefficient estimates, which shows the extent of the confounding amongst the effects, revealed that there was 42 pairs of effects in this model with non-zero correlations. Since one or more of the pairs is greater than or equal to 0.5, there will be some difficulty separating the effects from each other when analyzing the data taking into consideration that confounding is normal in mixture designs. The equation of the fitted model is

Droplet size = −62.2901 X_1_ + 103.444 X_2_ + 92.3094 X_3_ + 1337.7 X_1_X_2_ + 1167.31 X_1_X_3_ + 112.612 X_2_X_3_ − 2582.93 X_1_X_2_X_3_ + 792.224 X_1_^2^X_2_^2^ + 766.274 X_1_^2^X_3_^2^ + 7.99058 X_2_^2^X_3_^2^

The combination of factor levels which minimizes the particle size and achieve a droplet size of 92.32 nm was found to be 15%, 10% and 75% for oil, surfactant and cosurfactant, respectively. These values indicate that minimum globule size could be achieved at low oil and surfactant levels and high cosurfactant%. The SNEDDS formulation that contains these levels was prepared and characterized and the observed value for the particle size and PDI were found to be 94.43 ± 3.55 nm and 0.544, respectively.

### 3.2. Characterization of Pastes

The solid content in the prepared paste formulations constituted more than 50% of the total weight, which account for the reduction in tablet weight that occurred during the drying process. The change in tablet weight, for each formulation, before and after drying is illustrated in Table 2. This behavior is predictable since wet tablets undergo significant shrinkage upon drying as previously reported [9,38]. In this work, initial screening of the avicel, lactose and PVP percentages was conducted. Zidan et al. studied 10–30% *w*/*w* and 13–60% *w*/*w* of lactose and avicel, respectively, during 3D-printing of modified release tablets containing diclofenac sodium [38]. They reported that pastes containing high content of avicel (about 60% *w*/*w*) required high extrusion pressure. Also, they mentioned that the aqueous solubility, hygroscopicity and/or swelling of the excipients are parameters that affect the 3D printing process. During the printing process, formulations prepared using low concentrations of avicel, PVPK90, lactose and HPMC resulted in pastes that were not suitable for printing due to low viscosity and consistency, while higher concentration of these polymers, especially for avicel and PVPK90, resulted in a very viscous pastes that were difficult to print since they required higher extrusion pressure. This finding is in a good agreement with a previous work wherein researchers tested the extrudability of carbopol based 3D printing pastes [38].

All of the studied pastes were developed into 3DP tablets after extrusion from 0.58 mm nozzle at an optimum flow speed of 2.6 mm/s. Initial screening of the studied polymer concentrations and the processing parameters were carried out to predict the optimum condition for printing. Changing the flow speed to a lower value did not allow continuous deposition of the tablet layers, while a higher flow speed did not permit good construction of intact tablet layers due to insufficient drying time for the printed layer. The studied nozzle size was selected to allow free flowing of the pastes at the adjusted flow speed. Previous reports indicated that the nozzle size affects the flow index rather than the consistency of the paste. Rahman et al. and Zhang et al. mentioned that a narrow orifice resulted in a low flow rate, which increased the applied flow pressure, while a wider nozzle orifice (0.6 mm) enhanced the flow rate [39,40].

**Figure 1:**
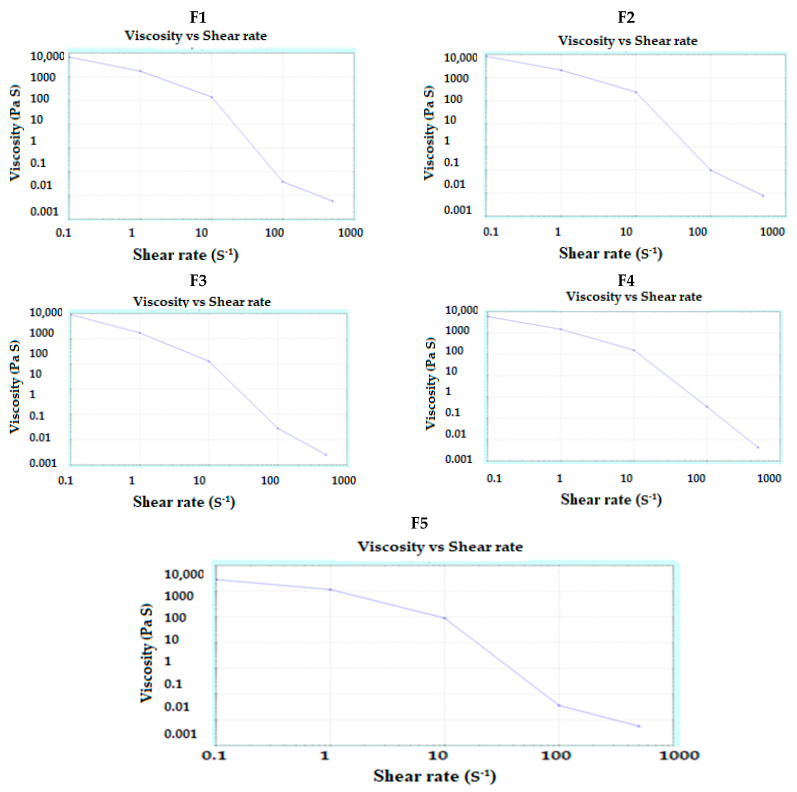
Rheograms of the prepared pastes (**F1**–**F5**) used in 3D-printing.

Rheological characterization of the prepared pastes indicated that the studied pastes exhibited shear-thinning behavior (Figure 1). All the studied pastes exhibited a pseudoplastic behavior with a value of flow index “n” less than 1. There was a decrease in the apparent viscosity upon increasing the shear rate. This behavior is expected to prevent clogging of the nozzle and overfilling of the formulation at specific locations where there is a change of the direction during the printing process. The rheograms obtained are in accordance with previous work [41]. It must be mentioned that during the printing process using the REGEMAT3D V1 BioPrinter, the applied pressure was kept constant and only the flow rate was adjusted to allow continuous deposition of the layer during the extrusion process. Viscosity values of the prepared pastes are represented in Table 2. The obtained results for the rheological behavior of the prepared pastes are in a good agreement with a previous work for carbopol based 3DP pastes [38]. SNEDDS-based pastes illustrated lower viscosity value than the corresponding non-SNEDDS pastes. This could be attributed to the presence of SNEDDS components (oil, PEG and tween 80) that lubricate the solid particles and prevent water loss. SNEDDS and non-SNEDDS pastes exhibited almost similar flow and rheological behavior except for slight differences that could be attributed to the existence of SNEDDS in the former as illustrated in Figure 1**.** Addition of the studied drug(s) insignificantly affected the viscosity value.

### 3.3. Characterization of 3DP Tablets

**Figure 2:**
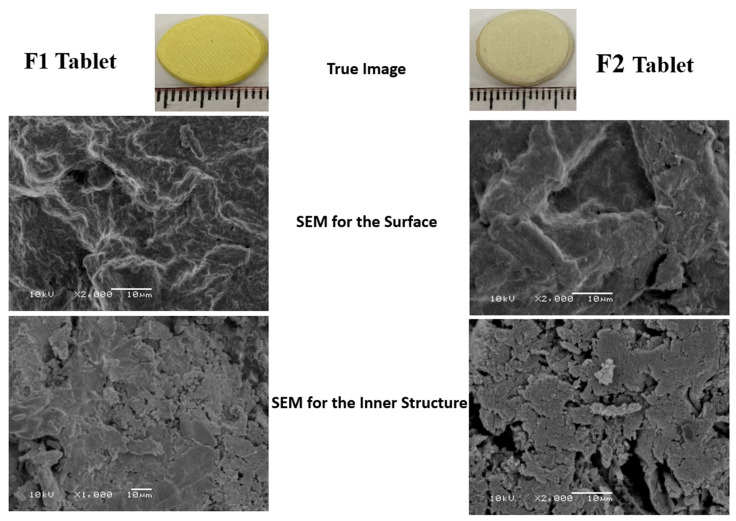
Images and SEM photographs for the surface and inner structure of SNEDDS-based (**F1**) and non-SNEDDS (**F2**) based 3DP tablets.

Images for the dried tablet formulations containing either SNEDDS-based paste (F1) or non-SNEDDS paste (F2) are illustrated in Figure 2. The dried tablets maintained their original shape except for some decrease in the thickness and weight, and a minimum change in the diameter due to water evaporation that leads to shrinkage in the prepared tablets. Table 2 depicts the weight, thickness, diameter, friability, and drug content of the prepared dried tablets. Friability was less than 1%, which is an indication of a good mechanical strength. Tablets were prepared using 4 mg and 10 mg of GLMP and RSV, respectively. The dried tablets showed a drug content of 3.918 ± 0.219–4.052 ± 0.087 mg and 9.691 ± 0.594–9.76 ± 0.359 mg of GLMP and RSV, respectively. Tablets of formulation F6 were prepared using a combination of GLMP and RSV. The concentration of each drug in this formulation can be changed according to the personal need by changing the amount of the paste (number of layers) used during tablet printing.

**Figure 3:**
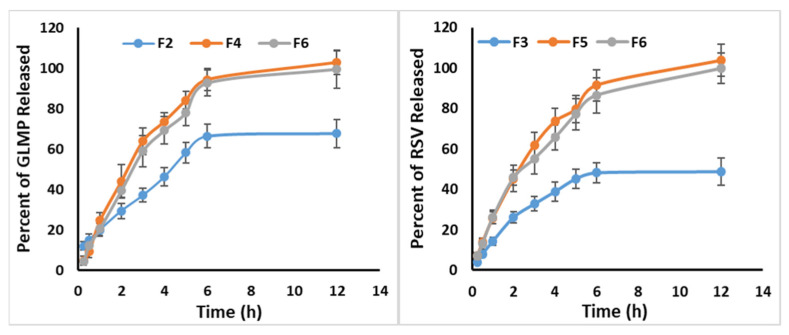
In vitro drug release of glimepiride and rosuvastatin from the prepared 3DP tablet formulations.

The in vitro release profiles of the prepared tablet formulations (F2–F6) are represented in Figure 3. All the studied formulations showed an extended drug release behavior. The release from the drug loaded SNEDDS-based tablet formulations was superior to that of the corresponding non-SNEDDS tablets. This effect could be attributed to the small size of the drug loaded SNEDDS in the GLMP formulations (F4 and F6) and RSV formulations (F5 and F6) which provided large surface area for the release of both drugs.

### 3.4. Solid-State Physicochemical Characterization

**Figure 4:**
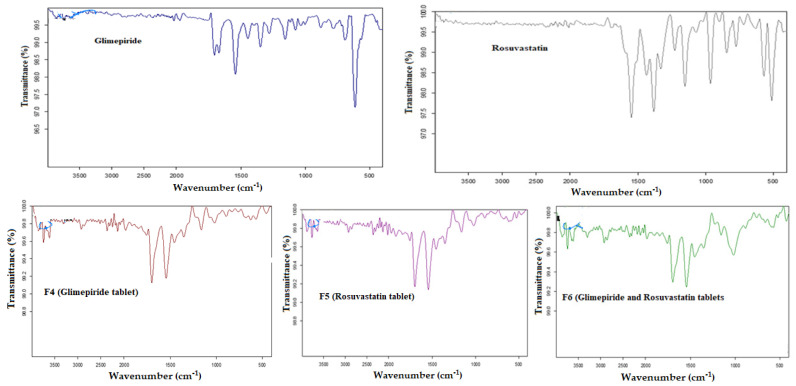
FT-IR spectra of glimepiride, rosuvastatin, and the prepared 3DP tablets.

The FT-IR spectrum of GLMP (Figure 4) demonstrated two characteristic peaks of the carbonyl (C=O) stretching which were detected at 1708 and 1674 cm^−1^. Another peak for C–N stretching vibrations was detected at 1344 cm^−1^. The sulfoxide (S=O) stretching vibration was noticed at 1151 cm^−1^. The FT-IR spectrum of RSV displayed a peak at 1550 cm^–1^ corresponding to C=C stretching, and a peak at 1515 cm^–1^ that is attributed to N–H bending. Peaks for the asymmetric and symmetric bending vibrations of the drug CH3 group were detected at 1485 cm^–1^ and 1380 cm^–1^, respectively. The asymmetric vibration of the sulfoxide (S=O) group was detected at 1330 cm^–1^. The bending vibration for C–H was detected at 1230 cm^–1^ while, the C–F stretching vibration was noticed at 1155 cm^–1^. The FT-IR spectrum of the formulated tablets showed disappearance for some of the characteristic GLMP peaks in F4 and F6, and RSV peaks in F5 and F6. This behavior could be attributed to internalization of GLMP and/or RSV in the SNEDDS before development of the 3DP tablets. Similar behavior was previously reported for RSV during development of drug loaded nano-porous structure [42]. It was also noticed that there was a reduction in the intensity of the characteristic drug peaks, in the studied tablets, due to low drug content in the formulated tableted when compared to pure drug spectra. This finding was previously reported by Kapure et al. during development of liquisolid tablets for improving dissolution of poorly water-soluble drug [43].

**Figure 5:**
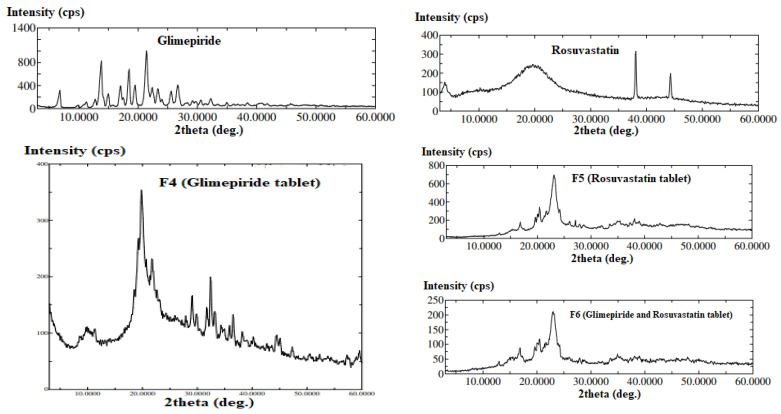
X-ray diffraction patterns of glimepiride, rosuvastatin, and the prepared 3DP tablets.

Figure 5 shows the XRPD of the pure drugs and the prepared tablet formulations. X-ray diffraction pattern demonstrated the crystalline nature of GLMP and RSV as both drugs displayed sharp distinct peaks at different deg. XRPD of the prepared tablet formulations showed marked changes in the pattern which is an indication of polymorphic changes upon loading of each drug in the SNEDDS system and development of 3DP tablet. Similar behavior was reported for liquisolid tablets and authors attributed this finding to solubilization of the drug in the liquid vehicle that was adsorbed onto the studied carrier and coating material of the liquisolid tablet [43]. This finding was also mentioned for leflunomide liquisolid tablets loaded with self-emulsifying drug delivery system [44].

### 3.5. Scanning Electron Microscope (SEM)

SEM images of SNEDDS and non-SNEDDS formulations revealed that no crystals were observed on the tablet surface (Figure 2). The surface of both tablet formulations shows low porosity and exhibited some curvature with the appearance of tortuosity that may have occurred during fusion and solidification of the layers. The surface of the SNEDDS-based tablet formulation was more homogenous and less porous which may be attributed to the presence of the SNEDDS formulation that render the paste consistent and decreased the rate of water evaporation during the drying step. The inner structure of F2 tablet showed dried interlocking flakes like structure with some void spaces and pores, as previously reported for HPMC based sustained release matrix tablets [45]. F1 tablet formulation illustrated a gel like structure with a smaller number of void and pores which may be due to the incorporation of the SNEDDS that makes the matrix wet and decreased the rate of water loss.

### 3.6. Pharmacokinetics Study

**Figure 6:**
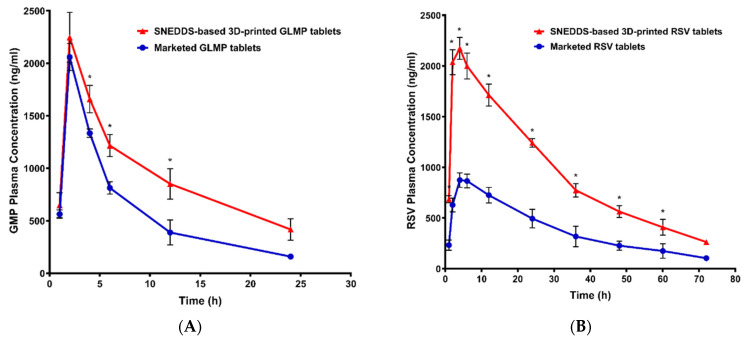
Plasma level-time curves for glimepiride (**A**) and rosuvastatin (**B**) after oral administration of the 3DP and marketed tablets to Male Wistar rats (n = 6). Note: * Indicates significant difference, at *p* < 0.05, between groups.

The calculated pharmacokinetic parameters for GLMP and RSV from the prepared 3DP tablets (F6) and the marketed drug products are illustrated in Table 3. The plasma level time curves of GLMP and RSV after oral treatment are represented in Figure 6. The obtained results indicated the superiority of the prepared 3DP tablets in the rate and extent of absorption. GLMP and RSV showed higher maximum plasma concentration over the time span from the 3DP tablets when compared to the marketed products. This effect is attributed to the existence of each drug in the nano-sized form (SNEDDS) which enhances the drug absorption from the gastrointestinal tract as previously reported for vitamin K from self-nanoemulsifying lyophilized tablets [46]. The time to reach the maximum plasma drug concentration was obtained after 2 ± 0 and 4.67 ± 1.15 for GLMP and RSV, respectively in all the studied tablets due to administration of the prepared and the marketed tablets to the studied animals as a suspension. The area under the plasma level-time curve (AUC) and the area under the moment curve (AUMC) were higher in the 3DP formulation. The relative bioavailabilities, calculated as [(AUC test/AUC marketed product) × 100], were 159.50% and 245.16% for GLMP and RSV, respectively. The marked enhancement in RSV relative bioavailability (245.16%) when compared to GLMP (159.50%) could be attributed to the limited bioavailability of the former (about 20%). Administration of RSV as an oral tablet loaded with a SNEDDS formulation leads to enhancement in the drug dissolution and absorption which was reflected on its bioavailability. GLMP is characterized by its low aqueous solubility (1.6 µg/mL) and slow dissolution rate, an effect that leads to low and variable oral drug bioavailability [47,48]. Enhancement in GLMP dissolution is expected to have a positive impact on the drug relative bioavailability from the developed 3DP tablets. In general, animals given the 3DP tablets showed enhancement in the pharmacokinetic behavior when compared to the commercial tablets.

## 4. Conclusions

Curcumin oil was successfully extracted from the dried rhizomes of *curcuma longa.* Mixture experimental screening design has been utilized in the development of a SNEDDS formulation of droplet size equals 94.43 ± 3.55 nm utilizing tween 80 and PEG 400 as a surfactant and cosurfactant, respectively. HPMC gel pastes, with or without SNEDDS formulation, were used to develop 3DP tablets. Rheological characterization of the prepared pastes showed a pseudoplastic shear thinning behavior. Tablets contain GLMP and/or RSV were developed utilizing a 3D BioPrinter using 0.58 mm nozzle and a flow speed of 2.6 mm/s. The release of the studied drugs was higher from the SNEDDS containing tablets. The developed 3DP tablets loaded with RSV and GLMP showed improved pharmacokinetics behavior when compared to the marketed drug products. The concentration of GLMP and RSV in these tablets could be changed according to the personal need.

## Figures and Tables

**Table 1:** Composition and droplet size of the studied SNEDDS formulations according to the extreme vertices mixture design.

Run	X_1_ (%)	X_2_ (%)	X_3_ (%)	Droplet Size (nm)		PDI
				Observed	Fitted	
1	15.0	30.0	55.0	105.0	103.44	0.303 ± 0.018
2	15.0	10.0	75.0	93.0	92.31	0.398 ± 0.020
3	25.0	20.0	55.0	359.0	355.01	0.554 ± 0.033
4	25.0	10.0	65.0	313.0	306.84	0.667 ± 0.008
5	17.5	23.75	58.75	157.0	156.95	0.320 ± 0.020
6	17.5	13.75	68.75	144.0	140.92	0.323 ± 0.022
7	22.5	18.75	58.75	279.0	272.67	0.454 ± 0.009
8	22.5	13.75	63.75	251.0	254.19	0.554 ± 0.019
9	15.0	20.0	65.0	123.0	126.03	0.234 ± 0.030
10	20.0	25.0	55.0	233.0	238.56	0.421 ± 0.021
11	20.0	10.0	70.0	196.0	200.69	0.428 ± 0.012
12	25.0	15.0	60.0	295.0	305.94	0.664 ± 0.088
13	20.0	17.5	62.5	205.0	199.45	0.330 ± 0.021

Abbreviations: X_1_, curcuma oil; X_2_, tween 80; X_3_, Polyethylene glycol 400; PDI, Polydispersity Index.

**Table 2:** Composition and characteristics of the prepared pastes and the 3DP tablets.

	F1	F2	F3	F4	F5	F6
Drug	-	GLMP	RSV	GLMP	RSV	GLMP and RSV
Vehicle	SNEDDS in DW	DW	DW	SNEDDS in DW	SNEDDS in DW	SNEDDS in DW
Viscosity (Pa. s)	6550 ± 278	8604 ± 295	9185 ± 153	6648 ± 135	6684 ± 451	-
Weight before drying (mg)	1003.33 ±1.32	965.46 ± 46.55	983.75 ± 4.29	1053.53 ± 5.84	1017.14 ± 61.69	2095.11 ± 167.56
Weight after drying (mg)	532.58 ± 23.7	490.53 ±27.76	498.88 ± 5.32	541.68 ± 61.02	559.39 ± 41.45	1067.54 ± 77.32
Thickness (mm)	3.03 5± 0.038	2.647 ± 0.160	2.826 ± 0.228	3.239 ± 0.178	2.876 ± 0.128	5.797 ± 0.156
Diameter (mm)	14.656 ± 0.148	13.289 ± 0.485	15.201 ± 0.228	14.855 ± 0.319	14.082 ± 0.249	14.342 ± 0.109
Friability (%)	0.068	0.159	0.199	0.117	0.043	0.089
Drug content (mg)	-	3.98 ± 0.201	9.76 ± 0.359	3.918 ± 0.219	9.691 ± 0.594	4.052 ± 0.087 and 9.711 ± 0.097

Abbreviations: GLMP, glimepiride; RSV, rosuvastatin, SNEDDS, self-nanoemulsifying drug delivery system; DW, distilled water. Notes: The concentrations of GLMP and RSV in the prepared tablets were 4 and 10 mg, respectively. F6 was printed using equal amounts of F4 and F5.

**Table 3:** Pharmacokinetic parameters of glimepiride and rosuvastatin after oral administration to Male Wistar rats (n = 6).

Parameter	Unit	SNEDDS-Based 3D-Printed GLMP Tablets		Marketed GLMP Tablets		SNEDDS-Based 3D-Printed RSV Tablets		Marketed RSV Tablets	
		AVERAGE	STDEV	AVERAGE	STDEV	AVERAGE	STDEV	AVERAGE	STDEV
K	1/h	0.060412	0.012131	0.097177	0.015336	0.030944	0.001221	0.033122	0.004295
t½	h	11.75933	2.135468	7.247718	1.095357	22.42318	0.875258	21.18031	2.931922
Tmax	h	2	0	2	0	4.666667	1.154701	4.666667	1.154701
Cmax	ng/mL	2246.667	238.5211	2058.667	128.8112	2191	82.48636	888	54.06478
AUC 0-t	ng/mL × h	22365.33	2683.317	14021.67	1539.859	68940.67	4257.624	28120	3200.072
AUC 0-inf_obs	ng/mL × h	29643.17	5503.715	15688.86	1717.492	77473.63	4696.094	31334.14	3829.658
AUC 0-t/0-inf_obs		0.761104	0.057117	0.893852	0.014435	0.700672	0.002253	0.898177	0.01551
AUMC 0-inf_obs	ng/mL × h^2^	505737.5	169476.8	159519.7	24539.36	2572924	198656.9	1021568	217442.1
MRT 0-inf_obs	h	16.73354	2.834548	10.13952	0.700672	33.18806	0.655388	32.36328	3.440672
V_D_	(mg)/(ng/mL)	0.005731	0.000401	0.006671	0.000822	0.008361	0.000289	0.019567	0.002129
Cl	(mg)/(ng/mL)/h	0.000345	6.45 × 10^−5^	0.000643	7.04 × 10^−5^	0.000259	1.62 × 10^−5^	0.000645	8.48 × 10^−5^

Abbreviations: K, elimination rate constant; t½, elimination half-life; Tmax, the time point to reach the maximum plasma concentration; Cmax, the maximum plasma concentration over the time specified; AUC 0-t, the area under curve from zero time to the last measurable concentration; AUC 0-inf_obs, the area under the plasma concentration-time curve from time zero to infinity; AUMC0–inf, the area under the first moment of the plasma concentration-time curve was from time zero to infinity; MRT0–inf, the mean residence time; V_D_, the apparent volume of distribution; Cl, the total body clearance. Notes: AUC0–t was calculated by the linear trapezoidal method. AUC0–inf was estimated as the sum of the AUC0–t plus the ratio of the last measurable plasma concentration to the elimination rate constant. MRT0–inf was calculated from the ratio of AUMC to AUC. t½ was calculated as 0.693/K. Cl was calculated by dividing the dose by AUC. V_D_ was calculated by multiplying the total body clearance by MRT.

## Data Availability

Not applicable.
